# The coordinated action of RNase III and RNase G controls enolase expression in response to oxygen availability in *Escherichia coli*

**DOI:** 10.1038/s41598-019-53883-y

**Published:** 2019-11-21

**Authors:** Minho Lee, Minju Joo, Minji Sim, Se-Hoon Sim, Hyun-Lee Kim, Jaejin Lee, Minkyung Ryu, Ji-Hyun Yeom, Yoonsoo Hahn, Nam-Chul Ha, Jang-Cheon Cho, Kangseok Lee

**Affiliations:** 1 Department of Life Science, Chung-Ang University, Seoul, 06974 Republic of Korea; 2 Department of Agricultural Biotechnology, Seoul National University, Seoul, 08826 Republic of Korea; 3 Department of Biological Science, Inha University, Incheon, 22212 Republic of Korea

**Keywords:** Microbiology, Microbial genetics, Bacterial genetics

## Abstract

Rapid modulation of RNA function by endoribonucleases during physiological responses to environmental changes is known to be an effective bacterial biochemical adaptation. We report a molecular mechanism underlying the regulation of enolase (*eno*) expression by two endoribonucleases, RNase G and RNase III, the expression levels of which are modulated by oxygen availability in *Escherichia coli*. Analyses of transcriptional *eno-cat* fusion constructs strongly suggested the existence of *cis*-acting elements in the *eno* 5′ untranslated region that respond to RNase III and RNase G cellular concentrations. Primer extension and S1 nuclease mapping analyses of *eno* mRNA *in vivo* identified three *eno* mRNA transcripts that are generated in a manner dependent on RNase III expression, one of which was found to accumulate in *rng*-deleted cells. Moreover, our data suggested that RNase III-mediated cleavage of primary *eno* mRNA transcripts enhanced Eno protein production, a process that involved putative *cis*-antisense RNA. We found that decreased RNase G protein abundance coincided with enhanced RNase III expression in *E. coli* grown anaerobically, leading to enhanced *eno* expression. Thereby, this posttranscriptional up-regulation of *eno* expression helps *E. coli* cells adjust their physiological reactions to oxygen-deficient metabolic modes. Our results revealed a molecular network of coordinated endoribonuclease activity that post-transcriptionally modulates the expression of Eno, a key enzyme in glycolysis.

## Introduction

Genome-wide analyses of mRNA abundance at single-gene resolution have facilitated the identification of ribonuclease RNA targets in bacteria^1–7^. These studies showed that RNase III and RNase E endoribonucleases control the stability of mRNAs ranging from hundreds to thousands of genes, whereas RNase G, a paralog of the N-terminal catalytic domain of RNase E, affects the abundance of fewer mRNAs^1–4^. The C-terminal domain of RNase E, which is absent in RNase G, interacts with PNPase 3′ → 5′ exoribonuclease, RhlB RNA helicase, and the glycolytic enzyme enolase, forming multicomponent ribonucleolytic complexes termed RNA degradosomes^8–10^. In addition, it also acts as a negative modulator through binding to an inhibitor protein, RraA or RraB, at distinct sites^2,11–13^. In some cases, an RNA binding protein, Hfq, as well as small non-coding antisense RNAs, are associated with the RNA cleavage activity of RNase E (e.g. SgrS and *pts* mRNA, for a review see^14^).

A large body of evidence has been accumulated in recent years showing the importance of antisense RNA in the regulation of gene expression^6,15,16^. For instance, antisense RNAs regulate acid resistance^17,18^ and type I toxin-antitoxin production (for a review, see^19^) in *Escherichia coli*. The *Salmonella* AmgR/*mgtC* system has also been shown to be dependent on antisense RNA regulation^20^. In addition, RNase III, a highly conserved double-stranded RNA-specific endoribonuclease, has been shown to cleave target RNA transcripts that form intra-RNA molecular stem-loop structures by interacting with antisense RNAs^6,21,22^. This antisense RNA-mediated RNase III cleavage involves both *cis-* and *trans-*antisense RNAs transcribed from the same or different genomic loci of target genes, respectively^6,21^.

The single-strand-specific endoribonucleases RNase G and E are involved in the expression of genes encoding enzymes involved in the major carbon metabolism pathways. RNase E-deficient cells show reduced phosphoenolpyruvate carboxylase production, limiting the conversion of phosphoenolpyruvate to oxaloacetic acid^23^. RNase E also directly decays *ptsG* mRNA, which encodes the major glucose transporter, when the glycolytic pathway is blocked^24^. In addition, deletion of the *rng* gene, encoding RNase G, results in increased steady-state levels of *adhE*, *eno*, *glk*, *pgi*, and *tpiA* mRNAs, proteins that are involved in carbon metabolism^1,5,25,26^. Moreover, increased mRNA abundance of the *eno* and *tpiA* genes is directly associated with protein expression levels in *rng*-deleted cells^26,27^, while deletion of *cra*, encoding a catabolite repressor/activator, in *rng*-deleted *E. coli* cells also results in increased pyruvate production in the medium^28^. However, the mechanisms underlying these *rng*-mediated alterations in the levels of enzymes and products associated with carbon metabolism remain largely unknown.

While RNase E and G generally destabilise their target mRNAs, RNase III can both decay and process mRNAs. For instance, RNase III cleaves the 5′ UTR of target mRNAs to yield translationally active mRNAs^18,29,30^. It has also been suggested that many mRNAs can be stabilised by RNase III in *E. coli*, likely due to ribosomal protection of RNase III-processed mRNAs from ribonucleases^4^. The enzymatic activity of *E. coli* RNase III is known to be regulated through stress induced by entry into stationary phase, temperature and osmotic changes, and exposure to aminoglycosides^4,31–34^.

While investigating the molecular mechanisms underlying the negative regulation of *eno* gene expression by RNase G in *E. coli*, we observed that *eno* expression was also positively regulated by RNase III. Therefore, in this study, we examined the functional roles of RNase G and RNase III in *eno* expression, and characterised factors involved in this endoribonucleases-mediated regulation of *eno* expression.

## Results

### Effects of cellular concentrations of RNase III and/or RNase G on *eno* expression

**Figure 1:**
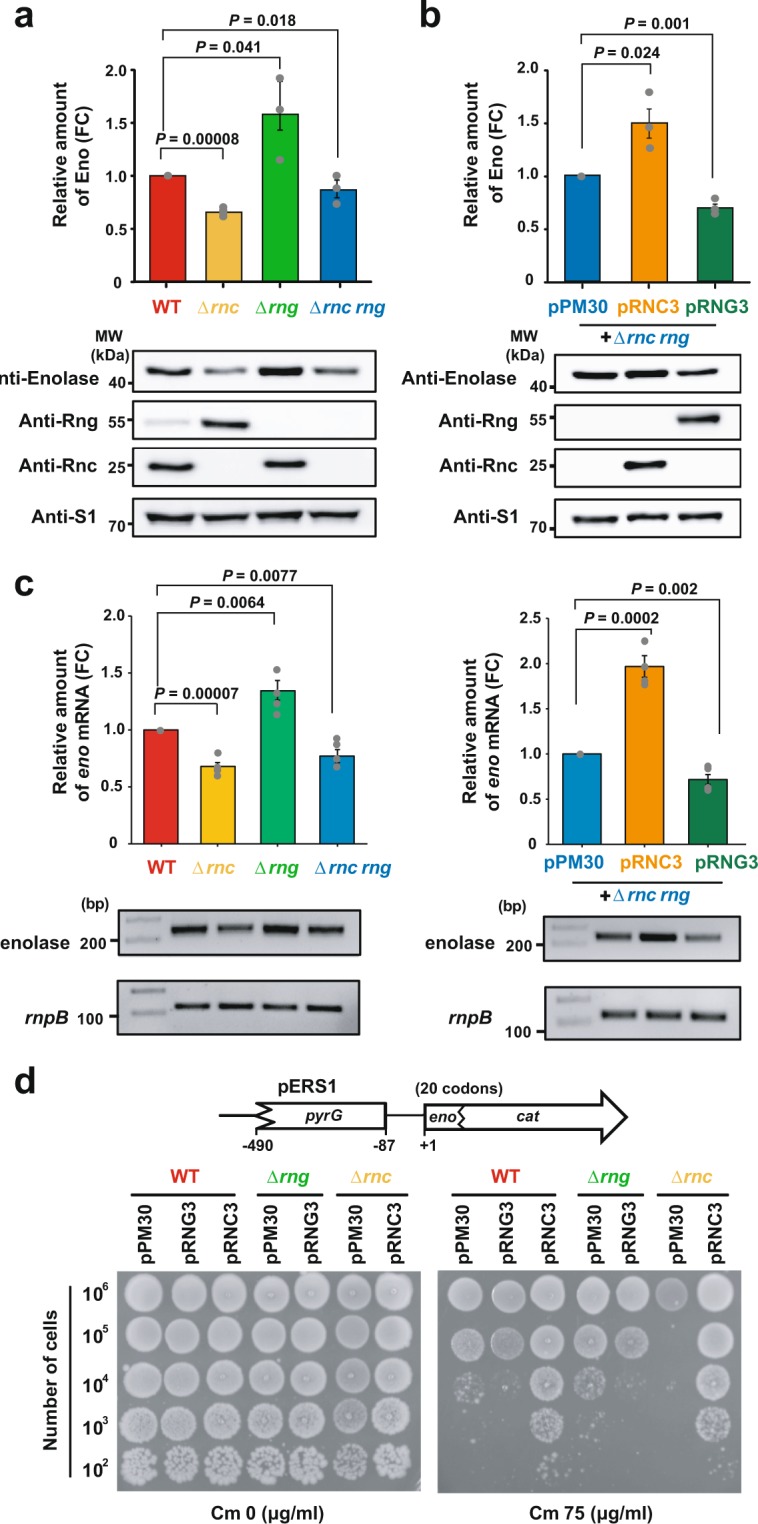
Regulation of Eno expression by RNase III and/or RNase G. **(a)** Effects of *rnc* and/or *rng* deletion on the expression level of *eno*. *Escherichia coli* MG1655 strains (WT, Δ*rng*, Δ*rnc*, and Δ*rnc rng*) were grown in LB medium at 37 °C to mid-log phase and harvested for western blot analysis of Eno, Rng, and Rnc using protein-specific polyclonal antibodies. The expression levels of Eno, Rng, and Rnc were compared by setting those of WT to 1. **(b)** Independent modulation of Eno expression levels by RNase G and RNase III. Western blotting was performed as described for **(a)** using Δ*rnc rng* strains harbouring pPM30, pRNG3, or pRNC3. The expression levels of Eno, Rng, and Rnc were compared by setting those of Δ*rnc rng* harbouring pPM30 to 1. **(c)** Effects of *rnc* and/or *rng* deletion on the *eno* mRNA abundance. Total cellular RNA was extracted from cultures grown to an OD_600_ of 0.6 using an RNeasy mini prep kit. The number of amplicons of *enolase* and other *rnpB* mRNA amplified from the cDNAs of the (left) WT, Δ*rnc*. Δ*rng* and Δ*rnc rng* strains (right) harbouring pPM30, pRNC3, or pRNG3. The *eno* mRNA expression levels were compared by setting those of WT or Δ*rnc rng* harbouring pPM30 to 1. PCR products were resolved in an 1.5% agarose gel. **(d)** Identification of the regulatory DNA region that affected the *eno* expression levels. Top: Schematic diagram of the *eno-cat* reporter. Bottom: Effects of RNase G and RNase III expression levels on the degree of chloramphenicol resistance of MG1655 cells. MG1655 WT, Δ*rng*, and Δ*rnc* cells harbouring pERS1 were transformed with pPM30, pRNG3 (RNase G), or pRNC3 (RNase III). The transformants were grown in LB containing 1 mM IPTG to an OD_600_ of 0.6, diluted, and spotted on LB agar containing 0 (Cm 0) or 75 (Cm 75) μg ml^−1^ chloramphenicol. For **(a,b)**, the S1 protein was used as an internal standard to evaluate the amount of cell extract in each lane. For **(c)**, the *rnpB* mRNA was used as an internal standard to evaluate the amount of cell extract in each lane. For **(a–c)**, the data are presented as means ± s. e. m. of at least three independent experiments.

RNase III has been shown to control *rng* mRNA stability by cleaving its coding region^34^. Therefore, we first tested whether RNase G-mediated down-regulation of *eno* expression is associated with RNase III by measuring *eno* expression levels in wild-type (WT), *rnc-* (Δ*rnc*), and/or *rng-*deleted (Δ*rng*) strains. Enolase expression increased approximately 1.6-fold in the Δ*rng* strain compared to that in the WT strain, as has been previously reported^27^ (Fig. 1a). Deletion of the *rnc* gene (Δ*rnc*) resulted in an approximately 30% decrease in *eno* expression. We reasoned that this decreased *eno* expression was due to increased expression levels of RNase G resulting from the stabilisation of *rng* mRNA in Δ*rnc* cells. Indeed, the expression levels of RNase G increased 8.8-fold in the Δ*rnc* strain compared to those in WT cells (Fig. 1a). However, inconsistent with the above results, we observed that *eno* expression levels decreased by approximately 15% in the *rnc* and *rng* double-mutant strain (Δ*rnc rng*); *eno* expression levels in the Δ*rnc rng* strain were expected to be similar to those in the Δ*rng* strain if RNase G alone is solely responsible for the posttranscriptional regulation of *eno* expression (Fig. 1b). The steady-state levels of *eno* mRNA were highly correlated with expression levels of Eno in the strains used in Fig. 1a–c). These results suggested the existence of RNase III-mediated positive regulation of *eno* expression independent of RNase G, in addition to the positive regulation of *eno* expression *via* destabilisation of *rng* mRNA.

The above results prompted us to test whether *eno* expression is directly regulated by RNase III cleavage *in vivo*. The Δ*rnc rng* strain was separately transformed with plasmids that can overexpress either RNase G (pRNG3) or RNase III (pRNC3), following which *eno* expression levels were assessed. As shown in Fig. 1b, RNase III overexpression in the absence of RNase G (Δ*rnc rng* + pRNC3) resulted in an approximately 67% increase in *eno* expression compared to the same strain harbouring an empty vector (pPM30), demonstrating the positive effect of cellular concentrations of RNase III on *eno* expression. Enolase expression was decreased by approximately 31% when RNase G was overexpressed in the Δ*rnc rng* strain (Δ*rnc rng* + pRNG3) (Fig. 1b). Enolase expression levels were not significantly affected by different cellular concentrations of RNase E, which plays a central role in mRNA decay in *E. coli* and presents substrate specificity and cleavage sites similar to those of RNase G (Supplementary Fig. 1).

### Identification of regulatory DNA region for *eno* expression

The above results indicate that Eno protein abundance is associated with cellular concentrations of both RNase G and RNase III. To determine whether the upstream region of the *eno* CDS contains the regulatory DNA region that responds to cellular RNase III and RNase G activity, we generated an *eno-cat* transcriptional fusion reporter construct (pERS1) expressing mRNA containing the 3′ end (403 nucleotides) of the *pyrG* coding sequence (CDS), the *eno* 5′ UTR (87 nucleotides), and the first 20 amino acids of the *eno* CDS (60 nucleotides) fused to the chloramphenicol acetyltransferase (*cat*) coding region (Fig. 1d). To compare the degree of chloramphenicol resistance among cells expressing different levels of RNase G and RNase III using this reporter system, MG1655 WT, Δ*rng*, and Δ*rnc* cells harbouring pERS1 were transformed with pPM30, pRNG3 (RNase G), or pRNC3 (RNase III), the replication origin of which is derived from pSC101^35^, thus compatible with pACYC177-derived pERS1. The results showed that, compared to the WT strain harbouring an empty vector (pPM30), overexpression of RNase G rendered the WT strain less resistant to chloramphenicol while overexpression of RNase III resulted in increased resistance to chloramphenicol (Fig. 1d). Overexpression of RNase G rendered the Δ*rng* strain more sensitive to chloramphenicol compared to the same strain harbouring an empty vector (Fig. 1d). In addition, when RNase III was overexpressed, the degree of chloramphenicol resistance of the Δ*rnc* strain was restored to that of the WT strain harbouring an empty vector (Fig. 1d). Collectively, these results showed a good correlation between the activities of the *eno-cat* fusion and the cellular concentrations of RNase G and RNase III (Fig. 1d), suggesting that the RNase G and RNase III-responsive regulatory DNA region is present between −490 to +60 region of the *eno* CDS.

We further characterised the RNase G and RNase III-responsive regulatory DNA regions by generating a pERS1-derived plasmid that did not contain the *pyrG* CDS (pERS2) and, using this reporter system, compared the degree of resistance to chloramphenicol among cells expressing different levels of RNase G and RNase III. We observed a good correlation between the activities of the *eno-cat* fusion and the cellular concentrations of RNase G and RNase III (Supplementary Fig. 2). The degree of chloramphenicol resistance in all the strains decreased because the removal of the DNA segment from the coding region of *pyrG* pERS1 resulted in a decreased synthesis of the primary *eno* transcripts (see below). These results suggest that RNase G and RNase III-responsive *cis*-acting elements were present between the −87 and +60 region of the *eno* CDS.

### Identification of RNase cleavage sites in *eno* mRNA

**Figure 2:**
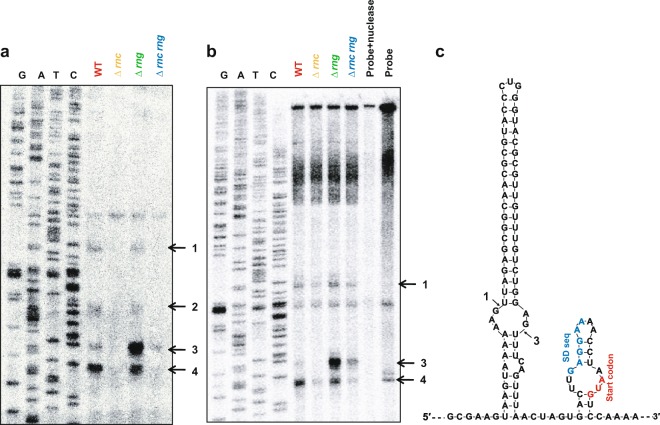
Identification of RNase cleavage sites in *eno* mRNA *in vivo*. **(a)** Primer extension analysis of the 5′ UTR of *eno* mRNA *in vivo*. Total RNA was isolated from MG1655 strains (WT, Δ*rng*, Δ*rnc*, and Δ*rnc rng*) and hybridised with the 5′ end ^32^P-labelled primer (eno + 52 R). Synthesised cDNA products were separated on a 6% polyacrylamide gel containing 8 M of urea. Sequencing ladders were synthesised with the same primers used for cDNA synthesis and PCR DNA encompassing the *eno* gene was used as a template. **(b)** S1 nuclease mapping. Total RNA was hybridised with the 5′ end ^32^P-labelled DNA probe. The DNA: RNA complex was treated with 1 U of S1 nuclease and separated in denaturing gel as described above. **(c)** Predicted *eno* 5′ UTR secondary structure and RNase cleavage sites. The secondary structure was inferred using the M-fold program. RNase III (1, 2, 3, and 4) cleavage sites identified in **(a)** and **(b)** are indicated. The putative Shine–Dalgarno sequence and start codon are indicated as blue and red colours, respectively.

The experimental results shown in Fig. 1d and Supplementary Fig. 2 raised a possibility that RNase III and/or RNase G regulate *eno* expression by cleaving the 5′ UTR of *eno* mRNA. To examine whether different *eno* mRNA species were generated in a manner dependent on the expression of RNase III and/or RNase G, we performed a primer extension analysis using total RNA extracted from the WT, Δ*rnc*, Δ*rng*, and Δ*rnc rng* strains and a 5′ end ^32^P-labelled primer (eno + 52R) designed to bind downstream of the N-terminal *eno* coding region. The results showed four cDNA bands (1, 2, 3, and 4) that were generated in a manner dependent on RNase III expression (Fig. 2a). When *eno* mRNA transcripts were further analysed by S1 nuclease mapping, three of the four putative cleavage products (1, 3, and 4) identified in the primer extension analysis were also detected (Fig. 2b). Canonical RNase III cleavage sites, characterised by a two nucleotide overhang at the 3′ end, were generated by cleavage sites 1 and 3 in the secondary structure of the 5′ UTR of *eno* mRNA predicted by the M-fold program (http://unafold.rna.albany.edu) (Fig. 2c). RNase III cleavage at sites 2 and 4 did not generate typical RNase III cleavage products. In addition, we observed a significant accumulation of band 3 in the *rng*-deleted strain in both the primer extension and S1 nuclease mapping analyses. These results suggest that RNase G degrades *eno* mRNA that is cleaved by RNase III at site 3. Putative cleavage sites 1 and 3 have been suggested as the 5′ ends of *eno* transcripts that were synthesised from two promoters (*enoP*4 and *enoP*6) in the intergenic region between *eno* and *pyrG*^36,37^. If products 1 and 3 were generated by RNase III cleavage, then the other closest transcription start site (*enoP*7) was located −211 base pairs (bp) from the start codon of the *eno* coding region, which explains why the 5′ end of the primary *eno* mRNAs was not detected in our primer extension and S1 nuclease mapping analyses.

To demonstrate biochemically the cleavage of *eno* mRNA by RNase III, an *in vitro* cleavage assay was performed using a model hairpin RNA of *eno* mRNA containing the RNase III cleavage sites (Supplementary Fig. 3). This synthetic RNA was labelled with ^32^P at the 3′ end to test for subsequent cleavage of RNase III-cleaved RNAs by RNase G. The results showed that RNase III cleaved the synthetic RNA at several sites that did not correspond to the sites identified by primer extension and S1 nuclease mapping analyses. We also observed that the intact synthetic RNA was not cleaved by RNase G, whereas RNA products generated by RNase III cleavage were cleaved by RNase G at the region encompassing the Shine–Dalgarno sequence of *eno* mRNA. These results indicated that other factors are involved in generating the RNase III cleavage products identified by the primer extension and S1 nuclease mapping analyses.

### Identification of candidate *cis*-antisense RNA and involvement of the corresponding chromosomal region in *eno* expression

**Figure 3:**
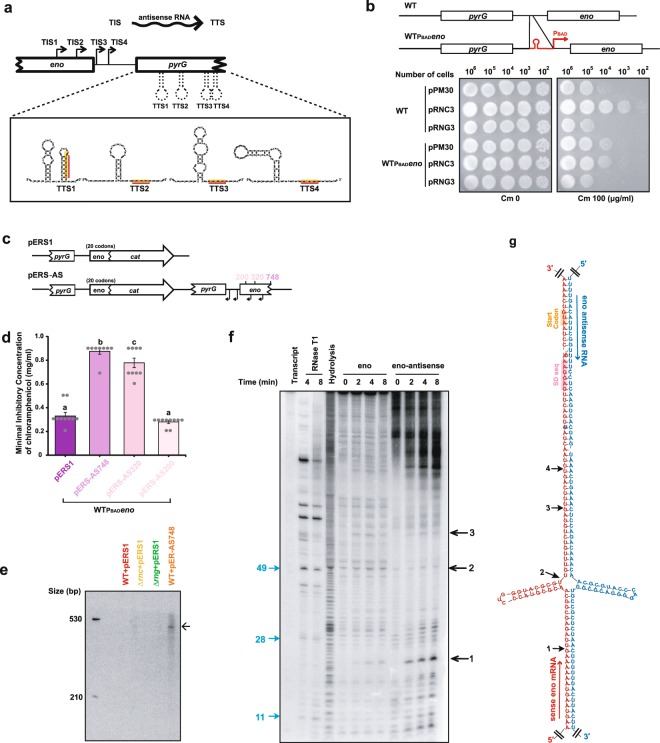
Restoration of RNase III-dependent regulation of *eno*-*cat* expression by putative *cis*-antisense RNA expression. (**a**) Schematic diagram showing the transcriptional initiation and termination sites (TISs and TTSs, respectively) of putative *cis*-antisense RNA. The 5′ and 3′ termini of putative *cis-*antisense RNAs that were inferred from cDNAs were located at positions +451, +272, −36, and −83 (designated as TIS1, TIS2, TIS3, and TIS4, respectively) from the start codon of the *eno* coding region, and at positions +1,419, +1,265, +736, and +696 from the start codon of the *pyrG* coding region (designated as TTS1, TTS2, TTS3, and TTS4, respectively). The secondary structure of the TTSs was inferred using the M-fold program. (**b**) Effects of alterations in the *eno* 5′ UTR on the regulation of *eno*-*cat* expression. Top: schematic representation of the region encompassing the *eno* and *pyrG* genes in W3110 WT and W3110_PBAD_*eno* strains. Bottom: degree of chloramphenicol resistance of W3110 and W3110_PBAD_*eno* strains harbouring pERS1. The W3110 and W3110_PBAD_*eno* strains harbouring pERS1 were transformed with pPM30, pRNG3 (RNase G), or pRNC3 (RNase III). The transformants were grown to an OD_600_ of 0.6 in LB containing 1 mM IPTG and 0.01% arabinose, diluted, and spotted on LB agar containing 0.01% arabinose and 0 (Cm 0) or 100 (Cm 100) μg ml^−1^ chloramphenicol. **(c)** Schematic representation of pERS1-derived plasmids that additionally contain a DNA segment encompassing the *eno* and *pyrG* genes (from + 875 of the *pyrG* coding region to + 748, + 320, or + 200 of the *eno* coding region). **(d)** Minimal inhibitory concentrations (MICs) of MG1655 harbouring pERS1, pERS-AS748, pERS-AS320, or pERS-AS200 against chloramphenicol. Measurements of the MICs were performed independently, in triplicate, in LB containing various chloramphenicol concentrations; significant differences are indicated with different letters (one-way analysis of variance [ANOVA] followed by the Student–Newman–Keuls test, *P* < 0.0001). **(e)** Northern blot analysis of putative *cis*-antisense RNA. Total RNA was isolated from the MG1655 strains harbouring pERS1or pERS-AS748 and used for northern blot analysis. **(f)** *In vitro* cleavage analysis of the half-length synthetic *eno* mRNA with or without *cis*-antisense RNA. The 5′ end-labelled *eno* transcript (1 pmol) was incubated with purified RNase III (1 pmol) in cleavage buffer with or without MgCl_2_ at 37 °C. Cleavage products were identified using size markers generated by alkaline hydrolysis and RNase T1 digestion. RNase T1 cleaves 3′ of single-stranded G nucleotides and cleavage products are indicated in blue bold characters. **(g)** The predicted secondary structure of *eno* 5′ UTR (red) and *cis*-antisense RNA (blue) encompassing RNase III cleavage sites. This secondary structure was inferred using the M-fold program.

*In vitro* cleavage assays using synthetic *eno* RNA and purified RNase III and RNase G did not generate the cleavage products identified by primer extension and S1 nuclease mapping analyses. Therefore, we suspected that other unknown factors were involved in RNase III- and/or RNase G-mediated cleavage of *eno* mRNA. First, we analysed cDNA from RNA-seq data of *E. coli* MG1655^38^ strain using an Artemis annotation tool^39^ to examine whether antisense RNA is involved in *eno* mRNA cleavage because RNase III can process mRNAs when duplexed with antisense RNAs^17,18^. Interestingly, a large number of cDNAs were found that appear to be synthesised from RNA species complementary to that of the *eno* mRNA 5′ UTR (Supplementary Fig. 4). These RNA species appeared to be synthesised from DNA segments encompassing the 5′ end of the *eno* and the 3′ end of the *pyrG* coding regions. The 5′ and 3′ ends of these antisense RNAs that were deduced from cDNAs were located at positions +451, +272, −36, and −83 (designated as TIS1, TIS2, TIS3, and TIS4, respectively) from the transcriptional start site of the *eno* coding region, and at positions +1,419, +1,265, +736, and +696 from the transcriptional start site of the *pyrG* coding region (designated as TTS1, TTS2, TTS3, and TTS4, respectively) (Fig. 3a and Supplementary Fig. 4).

To determine whether antisense RNA is involved in RNase III- and/or RNase G-mediated regulation of *eno* expression, we generated an *E. coli* strain containing alterations in the 5′ UTR of *eno* to prevent the production of putative antisense RNA(s) by inserting an exogenous DNA fragment (*P*_*BAD*_ promoter). This strain, designated W3110_PBAD_*eno*, was derived from strain TM447^24^ by removing the *cat* gene and replacing a segment of the intergenic region between the *eno* and *pyrG* genes (−45 to −28 from the *eno* coding region) with an arabinose-inducible promoter (*P*_*BAD*_) and a transcriptional terminator (Fig. 3b). In W3110_PBAD_*eno* cells, *eno* expression reached endogenous MG1655 cell levels when 0.001% arabinose was added to the culture (Supplementary Fig. 5).

The pERS1 plasmid was co-transformed with pPM30 (empty vector), pRNG3, or pRNC3 into W3110_PBAD_*eno* cells and the degree of chloramphenicol resistance was compared among the strains. RNase III expression-dependent *eno*-*cat* activity was lost in W3110_PBAD_*eno* cells harbouring pERS1 (Fig. 3b). Overexpression of RNase G still resulted in decreased *eno-cat* activity, implying that *eno*-*cat* mRNA synthesised in W3110_PBAD_*eno* cells serves as an RNase G substrate. These results indicate that the endogenous *eno* 5′ UTR has a role in RNase III-mediated regulation of *eno*-*cat* expression, possibly through *cis*-antisense RNA synthesised from the opposite DNA strand of the endogenous *eno* and *pyrG* genes.

### Restoration of RNase III-dependent regulation of *eno*-*cat* expression by putative *cis*-antisense RNA expression

The loss of RNase III-dependent regulation of *eno-cat* expression in W3110_PBAD_*eno* cells harbouring pERS1 (Fig. 3b) suggested that production of putative *cis*-antisense RNA from the opposite strand of the *eno* and *pyrG* genes was perturbed in the W3110_PBAD_*eno* strain. To test this possibility, we constructed a pERS1-derived plasmid that additionally contained a DNA segment from the *eno* and *pyrG* genes (from +875 of the *pyrG* coding region to +748 of the *eno* coding region) (Fig. 3c). The pERS-AS748 plasmid was transformed into W3110_PBAD_*eno* cells and the degree of chloramphenicol resistance of the resulting strains was compared. The results showed that pERS-AS748 could restore RNase III-dependent *eno-cat* expression in W3110_PBAD_*eno* cells, suggesting the ability of pERS-AS748 to express antisense RNA (Fig. 3d).

To characterise the main promoter and 5′ end of the putative antisense RNA, we constructed pERS-AS748-derived plasmids containing different DNA segments encompassing the *eno* and *pyrG* genes. These pERS-AS748 derivatives contained a DNA segment from +875 of the *pyrG* coding region to +320 (pERS-AS320) or +200 (pERS-AS200) of the *eno* coding region. These plasmids were co-transformed with pPM30, pRNG3, or pRNC3 into W3110_PBAD_*eno* cells and the resulting strains were tested for the degree of resistance to chloramphenicol (Fig. 3d). The results showed that pERS-AS320, but not pERS-AS200, could restore RNase III-dependent regulation of *eno-cat* expression in W3110_PBAD_*eno* cells, indicating that the promoter for antisense RNA expression was present in the region between +200 and +320 of the *eno* coding region, and that TIS2 was likely to be the transcriptional initiation site (Fig. 3d). Northern blot analysis of antisense RNA showed the existence of an RNA transcript approximately 500 nucleotides long in the WT strain harbouring pERS-AS748 (Fig. 3e). The transcript was not detected in the strains that harboured pERS1, probably because its endogenous level was low (Fig. 3e). Collectively, these results indicate that the putative *cis*-antisense RNA was synthesised from TIS2 and terminated at TTS1 (Supplementary Fig. 4).

### Effects of putative antisense RNA on RNase III cleavage activity on *eno* mRNA

To investigate whether the putative *cis*-antisense RNA is required for RNase III cleavage of *eno* mRNA, we performed an *in vitro* cleavage assay using purified RNase III, synthetic *eno* mRNA (−96 to +448 of the *eno* CDS), and *cis*-antisense RNA transcripts. RNase III-mediated cleavage of the 5′ end-labelled synthetic *eno* transcripts generated one major and several minor cleavage products in a *cis*-antisense RNA-dependent manner (Fig. 3e). One major band corresponded to cleavage product 1 (Fig. 3f,g, and Supplementary Fig. 6) that was identified in the primer extension and S1 nuclease mapping analyses of *eno* mRNA (Fig. 2a,b). Other bands (2–4 in Fig. 2a,b) were not readily identified in these *in vitro* cleavage assays (Fig. 3f). These results indicate a possible function of the putative *cis*-antisense RNA for *eno* mRNA cleavage by RNase III.

### Regulation of Eno expression by oxygen availability

**Figure 4:**
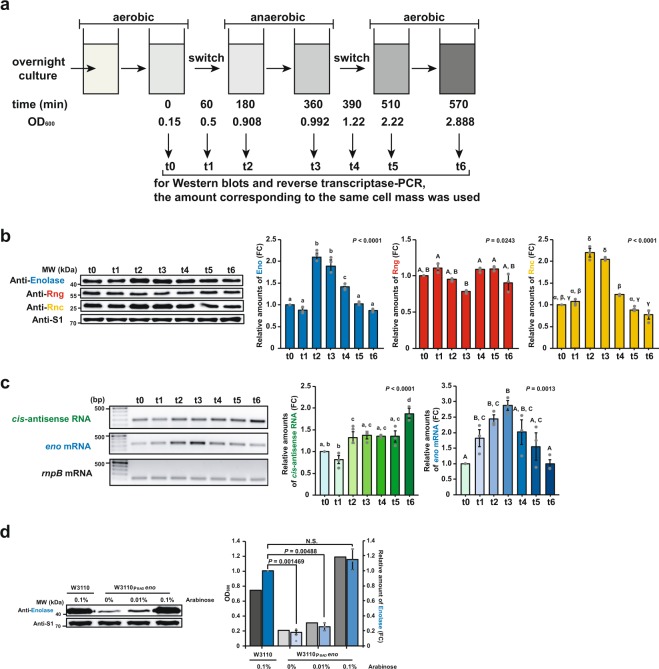
Expression levels of Eno, Rng, Rnc, and *cis*-antisense RNA depending on oxygen availability. **(a)** Schematic representation of the aerobic–anaerobic–aerobic alternating experiment. **(b)** Expression profiles of Eno, Rng, and Rnc depending on oxygen availability. WT MG1655 cells were cultured to each time point under aerobic or anaerobic conditions at 37 °C and then harvested for western blot analysis of Eno, Rng, and Rnc using protein-specific polyclonal antibodies. Blue, red, and yellow bars indicate the relative expression levels of Eno, Rng, and Rnc, respectively. The expression levels of Eno, Rng, and Rnc were compared by setting those of t0 to 1. Significant differences are indicated with different letters (one-way ANOVA followed by the Student–Newman–Keuls test; Small English letters indicate the difference from expression levels of Eno; Large English letters indicate the difference from expression levels of Rng; Greek symbols indicate the difference from expression levels of Rnc). **(c)** Analysis of *cis*-antisense RNA and *eno* mRNA expression in WT MG1655 cells using RT-PCR. The cDNA was synthesised from the total RNA extracted at each time point using the primers designed to bind *cis*-antisense RNA and *eno* mRNA. The PCR products were resolved in an 1.5% agarose gel. Significant differences are indicated with different letters (one-way ANOVA followed by the Student–Newman–Keuls test; Small English letters indicate the difference from expression levels of *cis*-antisense RNA; Large English letters indicate the difference of expression levels of *eno* mRNA). **(d)** Effect of Eno expression levels on the growth of W3110_PBAD_*eno* cells. Cultures of W3110_PBAD_*eno* cells were grown in LB medium containing 0.2% glucose under anaerobic conditions to the early log phase (OD_600_ = 0.05) and different concentrations of arabinose (0%, 0.01%, or 0.1%) were added to induce Eno synthesis. As a control, WT W3110 cells were grown in the same way described above and 0.1% arabinose was added. Cultures were further grown (5 h after induction) and were monitored by measuring the OD_600_. Cultures were harvested for western blot analysis of Eno using protein-specific polyclonal antibodies. The grey and blue bars indicate the OD_600_ values and the relative expression levels of Eno, respectively. The expression levels of Eno were compared by setting those of W3110 to 1. N.S., not significant. For **(b,d)**, the S1 protein was used as an internal standard to evaluate the amount of cell extract in each lane. For **(c)**, the *rnpB* mRNA was used as an internal standard to evaluate the amount of cell extract in each lane. For **(b**–**d)**, the data are presented as means ± s. e. m. of at least three independent experiments.

Previous studies have shown that Eno expression is up-regulated when *E. coli* cells are grown anaerobically^40–42^. For this reason, we hypothesised that Eno expression can be up-regulated when *E. coli* cells are grown anaerobically since glycolysis plays a major role in energy production in the absence of oxygen. To investigate whether oxygen availability affects the RNase-mediated regulation of *eno* expression, we measured the steady-state levels of Eno, RNase G, and RNase III in WT cells under aerobic–anaerobic–aerobic alternating conditions^43^ (Fig. 4a). Western blot analysis showed that, compared to WT *E. coli* cells grown under the initial aerobic conditions (t0), Eno expression levels increased by 1.9-fold after shifting to anaerobic conditions (t3), coinciding with a 2.0-fold increase and an approximately 22% decrease in RNase III and RNase G expression levels, respectively (Fig. 4b). The alterations in the expressions levels of these proteins were restored when the cultures reverted to aerobic conditions (t5) (Fig. 4b). Steady-state levels of *eno* mRNA were well correlated with Eno expression levels under aerobic–anaerobic–aerobic alternating conditions, whereas those of *cis*-antisense RNA increased continuously during anaerobic–aerobic switching growth conditions (t2–t6) (Fig. 4c). Therefore, Eno expression levels are modulated by RNase G and RNase III in response to oxygen availability.

We further investigated the effect of Eno expression levels on the growth of *E. coli* cells under low oxygen conditions by measuring growth yields of W3110_PBAD_*eno* cells depending on the Eno expression levels. Cultures of W3110_PBAD_*eno* cells were grown in Luria-Bertani (LB) medium containing 0.2% glucose under anaerobic conditions to the early log phase (OD_600_ = 0.05) and different concentrations of arabinose (0%, 0.01%, or 0.1%) were added to induce Eno synthesis. After 5 h, we measured the optical density (OD_600_) of the cultures and Eno expression levels in W3110_PBAD_*eno* cells. The results showed that Eno expression levels were very well correlated with growth yields (Fig. 4d). These results suggested that Eno expression can affect the growth of *E. coli* cells when they are grown anaerobically because glycolysis plays a major role in energy production in the absence of oxygen.

## Discussion

**Figure 5:**
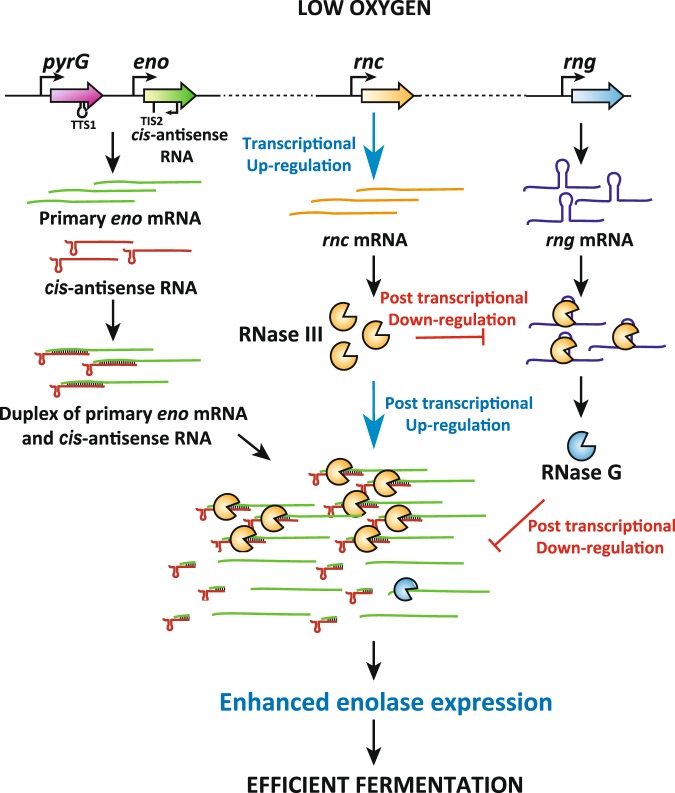
A model for the molecular mechanism involved in RNase III- and RNase G-mediated regulation of *eno* expression in response to oxygen availability in *E. coli*. RNase III and RNase G coordinately regulate *eno* expression in response to oxygen availability in *E. coli*. Low oxygen activates RNase III activity and promotes the degradation of *rng* mRNA, leading to decreased expression of RNase G. Enhanced RNase III-mediated cleavage of primary *eno* mRNA transcripts promotes Eno protein production under anaerobic conditions. This RNase III-mediated processing involves *cis*-antisense RNA synthesised from the *eno* coding region to that of *pyrG* in the opposite direction of mRNA synthesis of these genes.

Enolase is highly conserved in organisms from bacteria to humans^44^. It is an enzyme that catalyzes a reaction of glycolysis and also, known to be associated with several biological and pathophysiological processes^45–47^. The presence of enolase in the RNase E-based RNA degradation machinery regulates cell morphology in *E. coli* under anaerobic conditions^43^. The negative regulation of *eno* expression by RNase G at the posttranscriptional level has been previously shown in *E. coli*^1,27^. In this study, we identified additional regulatory pathways for *eno* expression that involve RNase III and *cis*-antisense RNA. Our findings imply that RNase III-mediated cleavage of sense-antisense *eno* mRNA is required for efficient *eno* mRNA translation. This enhanced *eno* mRNA translation by RNase III cleavage of the primary *eno* transcript might result from the removal of a large hairpin in the upstream of a putative Shine-Dalgarno sequence and the start codon (Fig. 2c), allowing ribosomes more accessible to these sites. In addition, *eno* expression is further enhanced by accelerated RNase III cleavage of *rng* mRNA through increased RNase III activity in *E. coli* cells grown anaerobically, thereby contributing to the adjustment of physiological reactions in *E. coli* cells to oxygen-deficient metabolic modes (Fig. 5). In this regard, it is worthwhile to note that the abundance of mRNAs encoding other glycolytic enzymes was affected by the cellular concentrations of RNase G^1^ (e.g. *glk*, *pgi*, and *tpiA*) and RNase III^4^ (e.g. *glk* and *ptsA*), implying that these endoribonucleases, expression levels of which are regulated upon oxygen availability, play an important role in modulating the expression level of glycolytic enzymes.

To understand the basis for the oxygen availability–dependent expression of the *rnc* gene, we analysed the promoter sequence. Bioinformatic analysis of these promoters (the online database Prodoric^48^) indicated the existence of sequences that were similar to the binding sequences for the oxygen-sensitive transcription factors fumarate nitrate reductase (FNR) and aerobic respiratory control (ArcA) in the promoter regions of *rnc* and *rng* (Supplementary Fig. 7). This complex regulatory system has been extensively studied in *E. coli*, where the DNA-binding proteins FNR and ArcA sense changes in oxygen availability and control the expression of many genes either alone or in cooperation with other regulators^42,49–53^. Further studies are required to characterise the molecular mechanisms for the regulation of RNase III expression in response to oxygen availability.

Although genome-wide analyses of RNase III cleavage sites implied the existence of *cis*-antisense RNAs that form duplexes with RNase III target mRNAs^5,6^. ArrS is the only *cis*-antisense RNA that has been experimentally suggested to have a role in sense mRNA processing^17,18^. Overexpression of ArrS resulted in the generation of *gadE* T3 mRNA, which contains a sequence complementary to ArrS in its 5′ UTR, leading to the production of more stable *gadE* T2 mRNA^17,18^. However, there is no direct experimental evidence showing ArrS-mediated RNase cleavage of *gadE* T3 mRNA. In the case of *eno*, genetic complementation and *in vitro* cleavage assays indicated the existence of *cis*-antisense RNA and its function in RNase III-mediated processing of the primary *eno* transcript (Fig. 3 and Supplementary Fig. 3). However, further studies are needed to unveil the detailed mechanisms underlying this endoribonucleases-mediated regulatory pathway for *eno* expression, including investigation of *cis*-antisense RNA biogenesis, sense-antisense RNA interactions, and enhanced translation of RNase III-processed *eno* mRNAs.

The involvement of both RNase III and RNase G in RNA function has been best characterised for 16S rRNA maturation in *E. coli*^26,54^. It was subsequently reported that incomplete processing of the 16S rRNA 5′ terminus by RNase G, which is down-regulated by increased RNase III activity on *rng* mRNA under aminoglycoside antibiotic stress, led to increased aminoglycoside resistance in *E. coli* cells^34^. A similar pathway involving RNase G and RNase III appears to be present in *Salmonella* Typhimurium^34^. These studies, together with our current study, suggest that bacteria commonly adopt endoribonuclease activity-mediated posttranscriptional regulatory pathways for rapid physiological adjustment to environmental changes.

## Methods

### Strains and plasmids

*E. coli* strains were grown at 37 °C in Luria-Bertani (LB) medium with or without 0.2% glucose supplemented with the appropriate antibiotics, under aerobic or anaerobic conditions. TM447 and W3110_PBAD_*eno* strains were cultured in the same medium supplemented with 0.001% L-arabinose^24^. For anaerobic growth of *E. coli* cells, a 30 ml cylindrical bottle containing a sterilised stir bar was fully filled with LB medium with 0.2% glucose, sealed with sealing tape, and then cultured on a magnetic stirrer^55^. For aerobic–anaerobic–aerobic alternating experiments, the cells were grown aerobically to OD_600_ ~0.15 (t0) as described above. The culture was subsequently shifted to anaerobic conditions and then returned to aerobic conditions. Aliquots of the cells at 0 (t0), 60 (t1), 180 (t2), 360 (t3), 390 (t4), 510 (t5), and 570 (t6) min were drawn for western blots and reverse transcriptase-polymerase chain reaction (PCR).

Both Δ*rnc* and Δ*rng* have been previously described^34,56^. To generate Δ*rnc rng*, the *rng* open reading frame was deleted in the Δ*rnc* strain using the procedure described by Datsenko and Wanner^57^. The primers used were: 5′-rng-KO (5′-GTGAGAAAAGGGATAAACATGACGGCTAATTGTTAGTAAACGTAACGGTGTAGGCTGGAGCTGCTTC-3′) and 3′-rng-KO (5′-TTACATCATTACGACGTCAAACTGCTCCTGGTTATAGAGCGGTTCAATATTCCGGGGATCCGTCGACC-3′), and pKD13^57^ was used as a template. To construct the pERS1 plasmid expressing the *eno-cat* fusion, PstI and BamHI sites were created by overlap-extension PCR using the following primers: Eno1 (5′-ATCTGCAGGCGGCCGCTGTGGCGCTGATTACCGAGT-3′), Eno2 (5′-TATCCAGTGATTTTTTTCTCAGTCGGGTTACCACGGGAGT-3′), Eno3 (5′-GAGAAAAAAATCACTGGATA-3′), and RMC-MscI (5′-CCTTGTCGCCTTGCGTATAA-3′). Two PCR products were obtained using primers Eno1/Eno2 and Eno3/RMC-Msc I and the PCR products were combined and amplified using the Eno1 and RMC-Msc1 primers. The resulting fragment was cloned into the pACYC177 using PstI and BamHI. To construct the pERS2 plasmid expressing the *eno-cat* fusion that doesn’t contain the *pyrG* CDS, overlap-extension PCR was performed using primers Eno4 (5′-ATCTGCAGGTAAAAAAGTTAGAGCGGCA-3′)/Eno2 and Eno3/RMC-MscI in the same manner as pERS1. The pRNC3 plasmid was constructed by subcloning the NotI and XbaI fragment from pRNC1 containing the RNase III coding region into the same sites in pPM30^4^. To express antisense RNA, a DNA fragment encoding a putative antisense *eno* RNA was amplified using the primers eno-nhe748R (5′-ATGCTAGCAAGCTGCGCAGTCCATCGCC-3′) and pyrG-nhe856F (5′-TAGCTAGCCCGGTAAGTGAAGTCACCAT-3′). The fragment was cloned into pERS1 using the NheI site, resulting in pERS-AS748. The pERS-AS320 and pERS-AS200 plasmids were constructed in a similar way, except that the 5′ end of the DNA segment was amplified using the eno-nhe 320 R primer (5′-TAGCTAGCCCGAATTTGGATTTGTTTTC-3′) for pERS-AS320 and eno-nhe200R (5′-GCGCTAGcGCTTTGGTTACGCCTTTACC-3′) for pERS-AS200. The mutant TM447 strain with a mutation in the *eno* 5′ UTR was obtained from Dr. H. Aiba^24^. The *cat* gene located upstream of *eno* in TM447 was removed using pCP20, resulting in W3110_PBAD_*eno*. The pRNG3 and pPM30 plasmids have been previously described^1^.

### Antibody purification and western blot analysis

Western blot analysis was carried out as previously described^4,58^. Polyclonal antibodies against Eno and RNase III were obtained from rabbits inoculated with purified His-tagged Eno and RNase III. Enolase and RNase III were purified from *E. coli* BL21(DE3) strains containing pET15b-enolase-His and pET15b-RNase III-His, respectively, using Ni-NTA agarose (Qiagen, Hilden, Germany). The proteins were eluted from columns using 125 mM imidazole and then concentrated and stored as previously described^59,60^. Polyclonal antibodies against RNase G, RNase E, and S1 were obtained from Dr. Stanley N. Cohen. Western blot images were obtained using an Amersham Imager 600 (GE Healthcare, Buckinghamshire, UK) and quantified using Quantity One (Bio-Rad Laboratories, Hercules, CA, USA). The ribosomal S1 protein was used as the control.

### Isolation of total RNA and reverse tanscriptase (RT)-PCR

Total cellular RNA was extracted from the cultures grown to an OD_600_ of 0.6 using an RNeasy mini prep kit (Qiagen). Following confirmation of the quality and quantity of the extracted total RNA with a Nanodrop 2000 instrument (Thermo Fisher Scientific, Waltham, MA, USA) the RT-PCR analysis was performed as previously described^61^. Briefly, cDNA was synthesised from 1 μg of total RNA using the PrimeScript 1st strand cDNA Synthesis Kit system for RT-PCR (Takara, Otsu, Japan) according to the manufacturer’s instructions. PCR primer sequences were designed according to the *eno, cis*-antisense RNA, and *rnpB* (as a standard control) genes in GenBank and products were obtained using primers, Eno RT F (5′-ATGTCCAAAATCGTAAAAAT-3′) and Eno RT R (5′- CATCTTTGCCAATCAGCGCC-3′) for *eno* mRNA; antisense RNA F (5′-TCACGGGAACCAGTAGAAGC-3′) and antisense RNA R (5′-TACGCGTTGTTTGTCTGGAG-3′) for *cis*-antisense RNA; and rnpB RT F (5′- TTGCTCCGGGTGGAGTTTAC-3′) and rnpB RT R (5′-GTGCAACAGAGAGCAAACCG-3′) for *rnpB* mRNA.

### Measurement of minimal inhibitory concentrations (MICs)

The MICs were measured as previously described^62^. Briefly, overnight cultures grown in LB medium supplemented with the appropriate antibiotics were diluted 1:100 in the same medium and incubated for 2 h. At an optical density 0.6 at 600 nm (OD_600_), 1 × 10^2^–10^6^ cells were spotted on LB agar plates containing different concentrations of antibiotics, or approximately 1 × 10^4^ cells were added to the same medium containing increasing concentrations of antibiotics. The cultures were grown for an additional 12 h and the lowest antibiotic concentrations that completely inhibited growth were designated as the MICs.

### Primer extension analysis

The procedure for primer extension analysis has been described previously^63^. Briefly, total RNAs were hybridised with a 5′ end ^32^P-labelled primer (eno + 60R: 5′-AGTCGGGTTACCACGGGAGT-3′) at 65 °C for 15 min, slowly cooled to 42 °C for 2 h, and then incubated at 42 °C for 1 h with AMV reverse transcriptase for cDNA synthesis (New England Biolabs, Ipswich, MA, USA). The amplicons were separated on 9% polyacrylamide gels containing 8 M urea and a sequencing reaction was performed and used as a molecular weight marker. Autoradiography was generated using a Packard Cyclone Phosphor Imager (PerkinElmer, Waltham, MA, USA) as previously described^64^.

### *In vitro* cleavage assay

Synthetic RNAs containing full-length and antisense *eno* sequences were synthesised from PCR DNA products using a MEGAshortscript T7 Kit (Thermo Fisher Scientific) according to the manufacturer’s instructions. The DNA products were PCR-amplified from MG1655 genomic DNA using the following primers: T7-eno −96F (5′-TAATACGACTCACTATAGGGCGAAGTAAGTAAAAAAGTT-3′), eno 448R (5′-TCGGAACCGGCATAGAGTAT-3′) for the full-length transcript, and T7-eno 273R (5′-CT TAATACGACTCACTATAGGGTCAATGCCAGCCTGATCTT-3′) and pyrG1153F (5′-CCACCTGCATACCCAGGCAA-3′) for antisense RNA. The RNAs were purified using a MEGAclear Kit and labelled at the 5′ end using [γ-^32^P] ATP. An aliquot (4 pmol) of ^32^P-5′ end labelled RNA was incubated with 1 ng of purified RNase III with 0.25 mg ml^−1^ yeast tRNA (Thermo Fisher Scientific), 20 U of RNase inhibitor (Takara), and RNase III cleavage buffer, with or without MgCl_2_ ^65^. Samples were removed at the indicated time points and separated on an 8% polyacrylamide gel containing 8 M urea.

### S1 nuclease mapping

Probes for S1 mapping were prepared by PCR amplification using the primers eno1 (5′-ATCTGCAGGCGGCCGCTGTGGCGCTGATTACCGAGT-3′) and eno10R (5′-TTTTGGACATTAGGTTTTCC-3′), after labelling of the 5′ ends of the primers with [γ-^32^P]ATP using T4 polynucleotide kinase at 37 °C for 1 h. The labelled *eno* 10 R primer and the unlabelled Eno1 primer generated a 500 bp *eno* probe. Total RNA (50 μg) was coprecipitated with 0.2 μg of the 5′ end ^32^P-labelled DNA probe and washed with 80% EtOH. The dried pellets were resuspended in 20 μl of hybridisation buffer and annealed at 52 °C overnight after denaturing at 80 °C for 10 min. After overnight incubation, S1 digestions were performed by adding 300 µl of an S1 nuclease mix (Promega, Madison, WI, USA) and incubating at 37 °C for 1 h. The protected fragments were electrophoresed alongside the sequence ladders obtained with the labelled primer used for probe preparation.

### Northern blot analysis

*E. coli* MG1655 (WT, Δ*rnc*, and Δ*rng*) cells harbouring pERS1 and MG1655 WT harbouring pERS-AS748 were grown at 37 °C to an OD_600_ of 0.6. Total RNA samples were prepared from the cultures using an RNeasy mini prep kit (Qiagen). Total RNA samples (40 µg) were denatured at 65 °C for 15 min in an equal volume of formamide loading buffer and separated by electrophoresis on a 1.2% GTG agarose gel containing 0.66 M formaldehyde. The gels were blotted onto a nylon membrane (Hybond-XL blotting membrane, Amersham) using Turboblotter (GE Healthcare). The pyrG1608F 5′ end ^32^P-labelled oligo probes (5′-CCCGCTGTTTGCAGGCTTTGTG-3′) were hybridised. The procedure for northern blot analysis was as previously described^1^. The size markers generated by internally labeled transcripts.

### Quantification and statistical analyses

All the statistical details of the experiments are included in the figure legends. Multiple comparison analysis was performed by the Student–Newman–Keuls test using SAS v.9.2 (SAS Institute, Cary, NC, USA), and the Student’s *t*-test was used for comparisons with controls using SigmaPlot (Systat Software, San Jose, CA, USA). The data are presented as means ± s. e. m., and *P-*values < 0.05 were considered significant^66,67^.

## Supplementary information

Supplementary information

## Data Availability

The datasets generated during and/ or analysed during the current study are available from the corresponding author on request.
