# Supplementary material for: The coordinated action of RNase III and RNase G controls enolase expression in response to oxygen availability in Escherichia coli
Source: Sci Rep. 2019 Nov 21;9:17257. doi: 10.1038/s41598-019-53883-y (PMC6872547; doi:10.1038/s41598-019-53883-y)

Supplementary Information

The coordinated action of RNase III and RNase G controls enolase expression in response to oxygen availability in *Escherichia coli*

Minho Lee[1†], Minju Joo[1†], Minji Sim[1†], Se-Hoon Sim[1†], Hyun-Lee Kim[1†], Jaejin Lee[1], Minkyung Ryu[1], Ji-Hyun Yeom[1], Yoonsoo Hahn[1], Nam-Chul Ha[2*], Jang-Cheon Cho[3*], and Kangseok Lee[1*]

[1]Department of Life Science, Chung-Ang University, Seoul 06974, Republic of Korea

[2]Department of Agricultural Biotechnology, Seoul National University, Seoul 08826, Republic of Korea

[3]Department of Biological Science, Inha University, Incheon 22212, Republic of Korea

*Corresponding authors: Nam-Chul Ha, e-mail: hanc210@snu.ac.kr, Tel: 82-2-880-4853, Fax: 82-2-873-5095; Jang-Cheon Cho, e-mail: chojc@inha.ac.kr, Tel: 82-32-860-7711, Fax: 82-32-232-0541; Kangseok Lee, e-mail: kangseok@cau.ac.kr, Tel: 82-2-820-5241, Fax: 82-2-825-5206

[†]These authors contributed equally to this work.

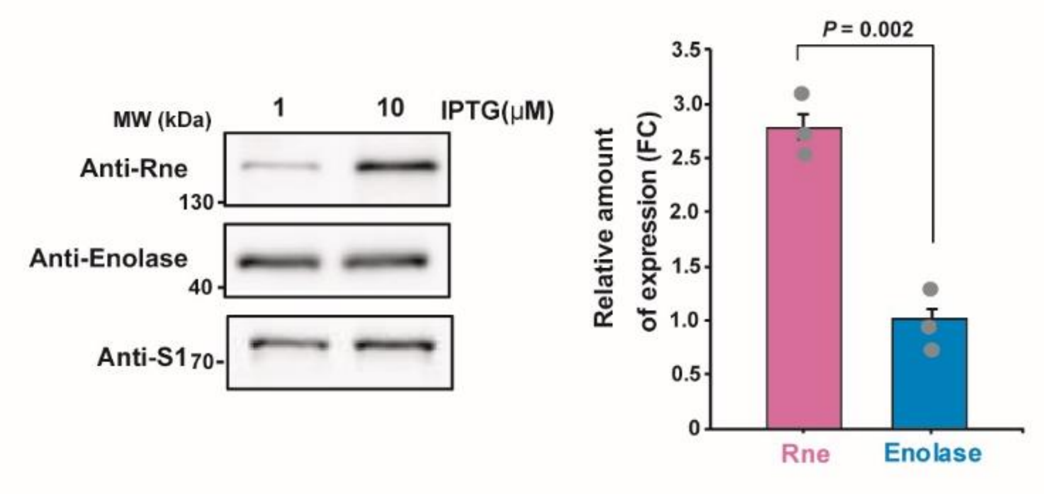

Supplementary Fig. 1. Effects of cellular concentrations of RNase E on enolase expression. In the *E. coli* KSL2003 strain[1] the synthesis of RNase E is controlled solely by the concentration of IPTG without significantly affecting normal cellular growth in LB medium. Steady-state levels of *eno* were measured in KSL2003 cells in 1 or 10 μM IPTG. Strains were harvested for western blot analysis of Eno and RNase E (Rne) using protein-specific polyclonal antibodies, and the S1 protein was used as an internal standard to evaluate the amount of cell extract in each lane. The data are presented as means ± s. e. m. of three independent experiments.

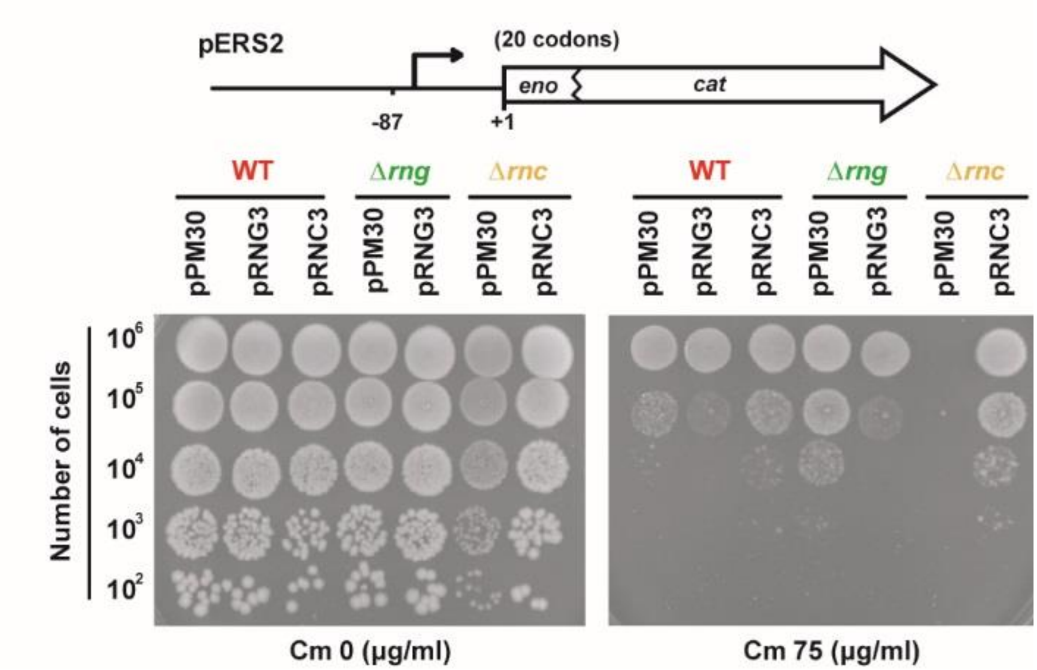

Supplementary Fig. 2. Identification of *cis*-acting elements that affect expression level of enolase.

Top: Schematic diagram of *eno-cat* fusion reporter. DNA segment encompassing the *eno* 5' UTR (87 nucleotides), and the first 20 amino acids of the *eno* CDS (60 nucleotides) was fused with CAT coding region. Bottom: Degree of chloramphenicol resistance in MG1655 cells expressing different levels of RNase G and RNase III. MG1655 WT, Δ*rng*, and Δ*rnc* cells harbouring pERS2 were transformed with pPM30, pRNG3 (RNase G), or pRNC3 (RNase III). The transformants were grown in LB containing 1 mM IPTG to an OD_{600} of 0.6, diluted, and spotted on LB agar containing 0 (Cm 0) or 75 (Cm 75) μg ml^{-1} chloramphenicol.

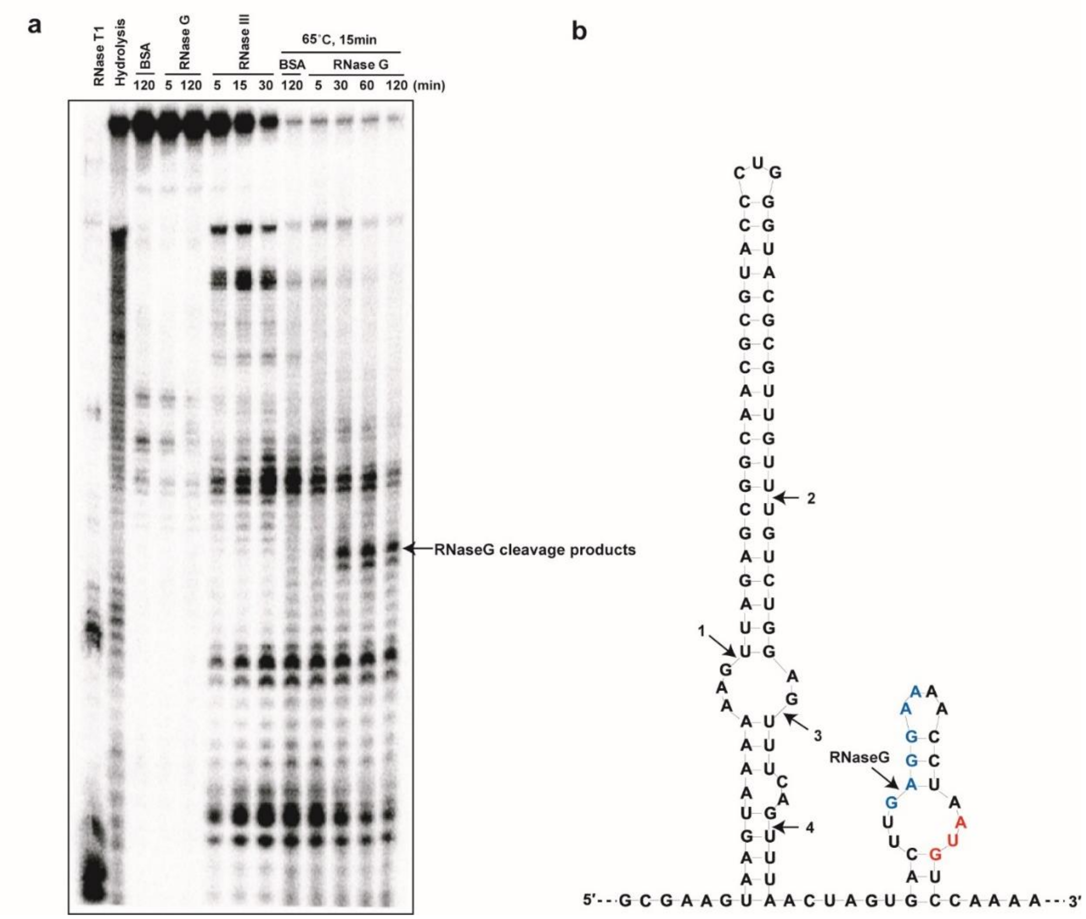

Supplementary Fig. 3. *In vitro* cleavage of *eno* mRNA by RNase III and RNase G.

(a) *In vitro* cleavage of *eno* mRNA. One picomole of 3' end-labelled *eno* mRNA was incubated with 0.5 μg of purified RNase G or RNase III at 37 °C. The cleavage reaction of RNase III was stopped at 30 min by incubation at 65 °C for 15 min. Then, the samples were incubated with 0.5 μg of purified RNase G at 37 °C for the periods shown on the top of the gel. The samples were withdrawn at the indicated time points and separated on an 8% polyacrylamide gel containing 8 M urea. The RNase G cleavage products are indicated (black arrows). **(b)** Predicted secondary structure of *eno* mRNA and RNase cleavage sites. The secondary structure was inferred using the M-fold program. RNase III cleavage sites (1, 2, 3, and 4) and RNase G cleavage sites identified in Fig. 2 and Supplementary Fig. 3a, respectively, are indicated. The putative Shine–Dalgarno sequence and start codon are indicated as blue and red colours, respectively.

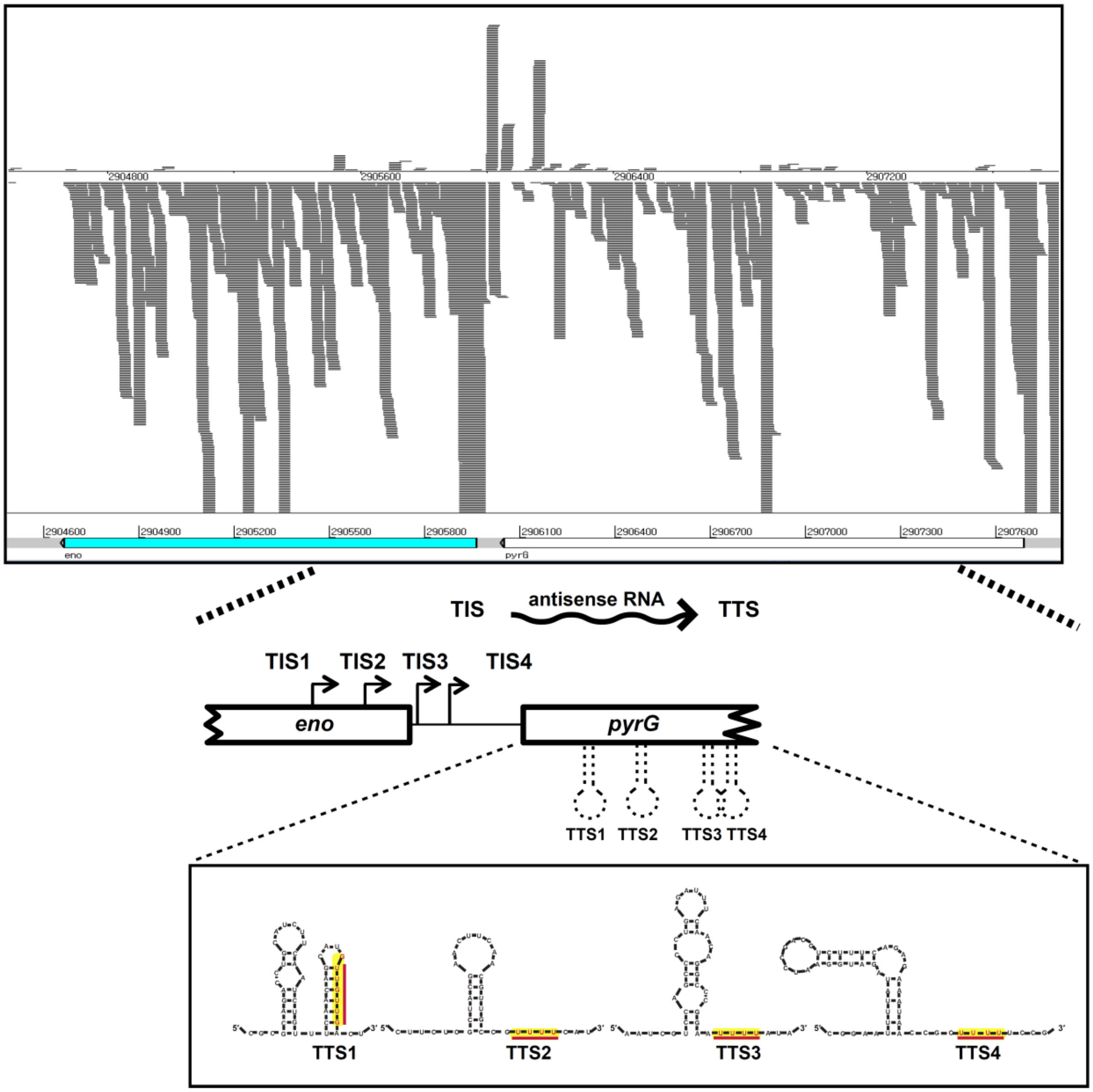

Supplementary Fig. 4. Characterisation of *eno* antisense RNA expression through bioinformatics analysis.

The diagram was generated by the Artemis program and shows cDNAs in the region between positions 2,904,600 to 2,907,750 in the genome of the *E. coli* MG1655 strain.

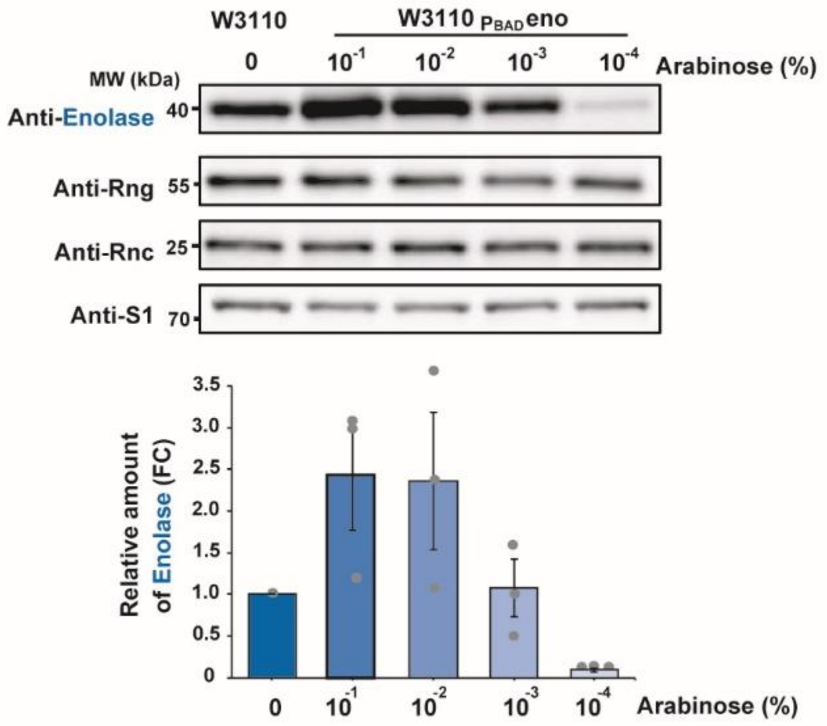

Supplementary Fig. 5. Expression of enolase in the *E. coli* W3110$_{\text{PBAD}}$*eno* strain.

Cultures of W3110$_{\text{PBAD}}$*eno* cells were grown in LB medium to the early log phase (OD_{600} = 0.1) and different concentrations of arabinose (1×10^{-1}, 10^{-2}, 10^{-3}, or 10^{-4}%) were added to induce Eno synthesis. Cultures were further grown to mid-log phase and were harvested for western blot analysis of Eno, Rng, and Rnc using protein-specific polyclonal antibodies. The expression levels of Eno, Rng, and Rnc were compared by setting those of WT to 1. The S1 protein was used as an internal standard to evaluate the amount of cell extract in each lane and the data are presented as means ± s. e. m. of at least three independent experiments.

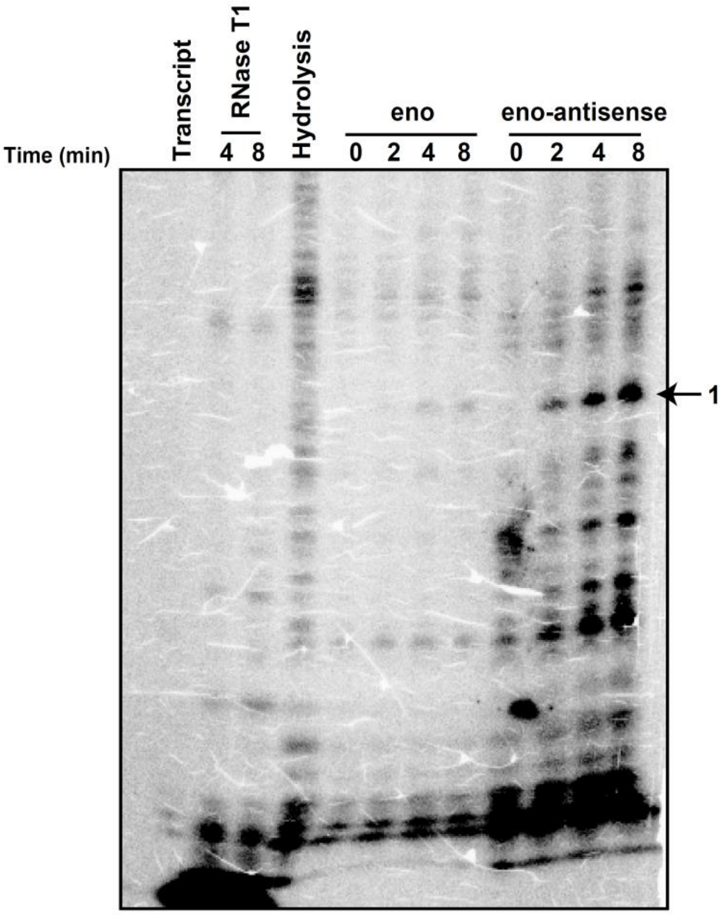

Supplementary Fig. 6. Identification of RNase cleavage sites in *eno* mRNA *in vivo.*

The same *in vitro* cleavage reactions used in **Fig. 3e** were separated on an 8% polyacrylamide gel containing 8 M urea with a shorter runtime than that used in **Fig. 3e** to visualise the 5′ end of the *eno* transcript.

a

Promoter Sequence

```
1     TTTCCTGGAT AAATTCCCTA AGACTAACGA CATCCCCTGT CGTTGTGTAT    50
51    AGAATATTCC CCCGAAGTTT TAGGTTGGCG CCGTTTGGTC GCCACGGCAC   100
101   ACGAAACAGC GTTGGTTATA GACAACCTTC TTTCCGCTGC AGCGATGCGG   150
151   CAGGACAGAT GACGTGTATC AGGTCTGTTT CGTGTGCTGG ATTGTTGACG   200
201   CATTCATTTA TTGGTATCGC ATG
```

PWM (Species)	Start Position	End Position	Strand	Score	Sequence
FNR - *Escherichia coli* (strain K12)	112	125	+	7.07	TTGGTTATAGACAA
FNR - *Escherichia coli* (strain K12)	112	125	-	7.04	TTGTCTATAACCAA
FNR - *Escherichia coli* (strain K12)	159	172	+	6.63	ATGACGTGTATCAG
FNR - *Escherichia coli* (strain K12)	159	172	-	6.51	CTGATACACGTCAT

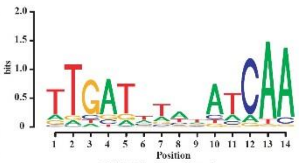

FNR Binding Site Logo

Weighted Homology Scores
- Maximum Possible Score: 8.93
- Minimum Possible Score: 0.40

Promoter Sequence

```
1     TTTCCTGGAT AAATTCCCTA AGACTAACGA CATCCCCTGT CGTTGTGTAT    50
51    AGAATATTCC CCCGAAGTTT TAGGTTGGCG CCGTTTGGTC GCCACGGCAC   100
101   ACGAAACAGC GTTGGTTATA GACAACCTTC TTTCCGCTGC AGCGATGCGG   150
151   CAGGACAGAT GACGTGTATC AGGTCTGTTT CGTGTGCTGG ATTGTTGACG   200
201   CATTCATTTA TTGGTATCGC ATG
```

PWM (Species)	Start Position	End Position	Strand	Score	Sequence
ArcA - *Escherichia coli* (strain K12)	114	123	+	5.75	GGTTATAGAC

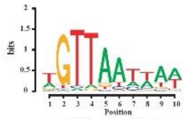

ArcA Binding Site Logo

Weighted Homology Scores
- Maximum Possible Score: 7.44
- Minimum Possible Score: 0.15

b

Promoter Sequence

```
1     AATGCTGCGT TTGCTTTCCG GAAATACGCA TCAGGTGATG ACTGCTGTTG    50
51    CGCTTGCGGA CTCGCAGCAG ACGCTGGATT GTCTGGTGGT AACGGAGGTG   100
101   ACTTTCCGCA CGCTTTCTGC GCAGGATATT ACTGGCTATG TCGCCAGCGG   150
151   CGAGCCTTTA GATAAAGCAG GTGCATACGG TATTCAGGGG CGGGGTGGCT   200
201   GTTTTGTCAG GAAGATAAAT GGCAGCTATC ACGCCGTGGT CGGCTTACCG   250
251   CTGGTTGAAA CGTATGAGTT GTTGAGTCAT TTTAACGCAC TGCGTGATAA   300
301   AAGGGATAAA CATG
```

PWM (Species)	Start Position	End Position	Strand	Score	Sequence
FNR - *Escherichia coli* (strain K12)	272	285	+	6.71	TTGAGTCATTTTAA
FNR - *Escherichia coli* (strain K12)	11	24	+	6.66	TTGCTTTCCGAAA

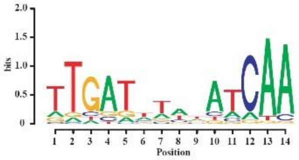

FNR Binding Site Logo

Weighted Homology Scores
- Maximum Possible Score: 8.93
- Minimum Possible Score: 0.40

Promoter Sequence

```
1     AATGCTGCGT TTGCTTTCCG GAAATACGCA TCAGGTGATG ACTGCTGTTG    50
51    CGCTTGCGGA CTCGCAGCAG ACGCTGGATT GTCTGGTGGT AACGGAGGTG   100
101   ACTTTCCGCA CGCTTTCTGC GCAGGATATT ACTGGCTATG TCGCCAGCGG   150
151   CGAGCCTTTA GATAAAGCAG GTGCATACGG TATTCAGGGG CGGGGTGGCT   200
201   GTTTTGTCAG GAAGATAAAT GGCAGCTATC ACGCCGTGGT CGGCTTACCG   250
251   CTGGTTGAAA CGTATGAGTT GTTGAGTCAT TTTAACGCAC TGCGTGATAA   300
301   AAGGGATAAA CATG
```

PWM (Species)	Start Position	End Position	Strand	Score	Sequence
ArcA - *Escherichia coli* (strain K12)	278	287	-	6.09	CGTTAAAATG
ArcA - *Escherichia coli* (strain K12)	270	279	+	5.67	TGTTGAGTCA

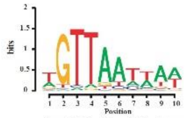

ArcA Binding Site Logo

Weighted Homology Scores
- Maximum Possible Score: 7.44
- Minimum Possible Score: 0.15

Supplementary Fig. 7. Analysis of binding of FNR and ArcA to *rnc* and *rng* promoters.

(a) Predicted FNR and ArcA binding sites in the promoter region of *rnc* using the online software Prodoric Virtual Footprint Promoter Analysis Version 3.0 online tool to predict *E. coli* K12 FNR and ArcA binding sites[2]. **(b)** Predicted FNR and ArcA binding sites in the promoter region of *rng* using the online software Prodoric Virtual Footprint Promoter Analysis Version 3.0 online tool to predict *E. coli* K12 FNR and ArcA binding sites. For **(a)** and **(b)**, FNR and ArcA binding sites are highlighted in black box, and a score indicating how closely the binding site

match FNR and ArcA binding sites consensus logo are given. A perfect match to the consensus sequence scores 8.93 and 7.44, respectively, as determined by Prodoric.

References

1 Lee, K., Bernstein, J. A. & Cohen, S. N. RNase G complementation of rne null mutation identifies functional interrelationships with RNase E in Escherichia coli. *Mol Microbiol* **43**, 1445-1456 (2002).

2 Eckweiler, D., Dudek, C. A., Hartlich, J., Brotje, D. & Jahn, D. PRODORIC2: the bacterial gene regulation database in 2018. *Nucleic Acids Res* **46**, D320-D326 (2018).

Blot Figure 4b

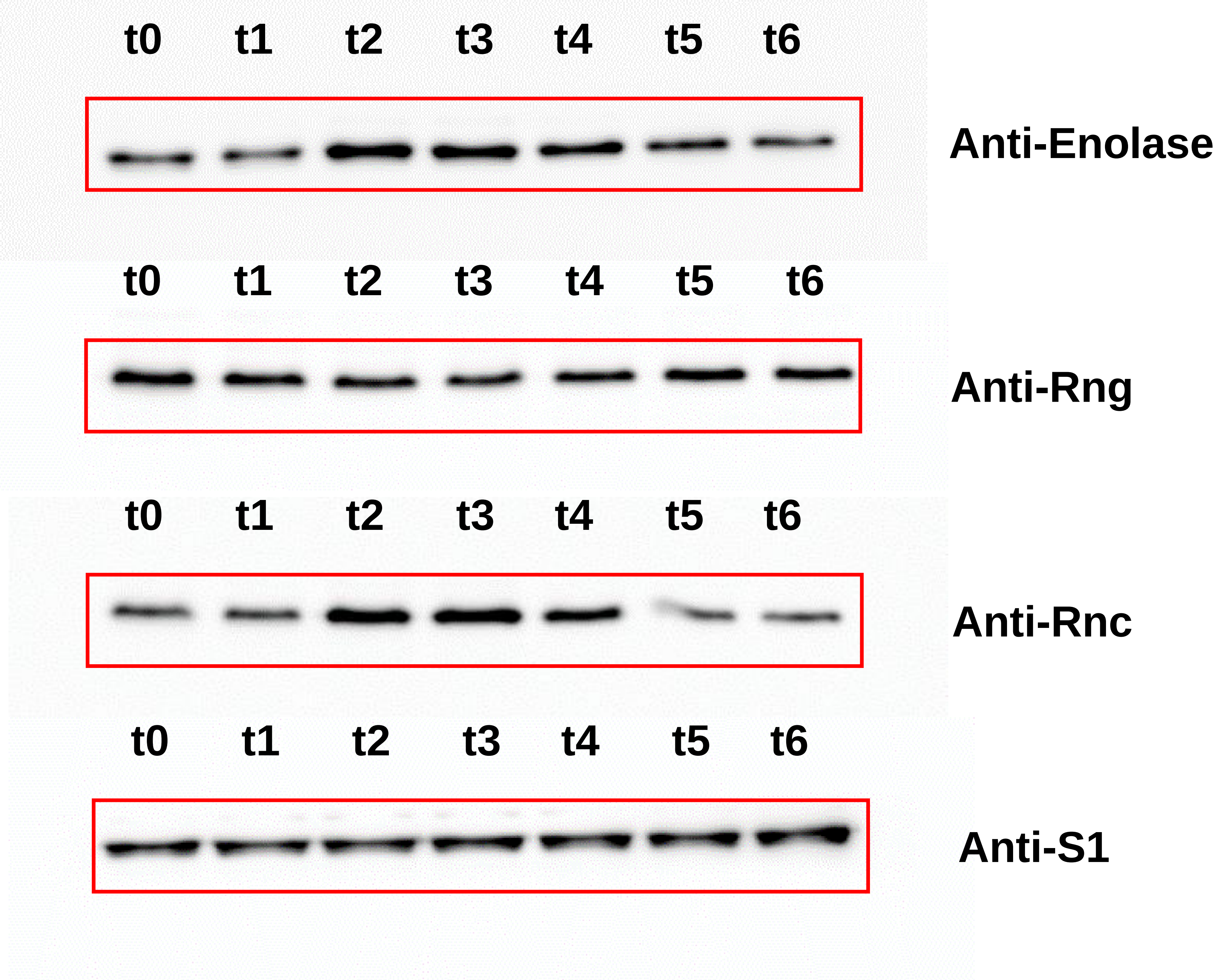

Gel Figure 4c

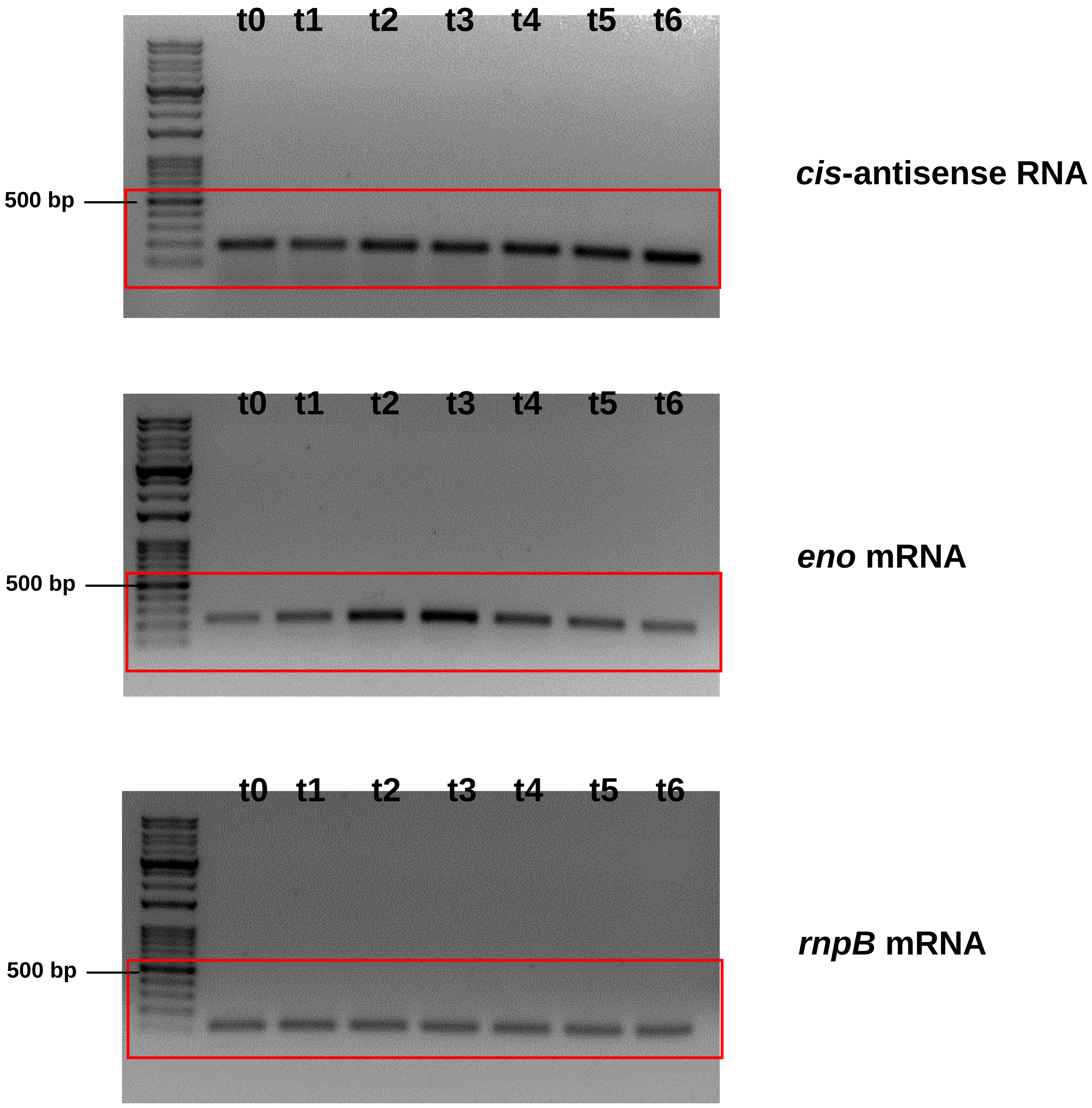

Blot Figure 4c

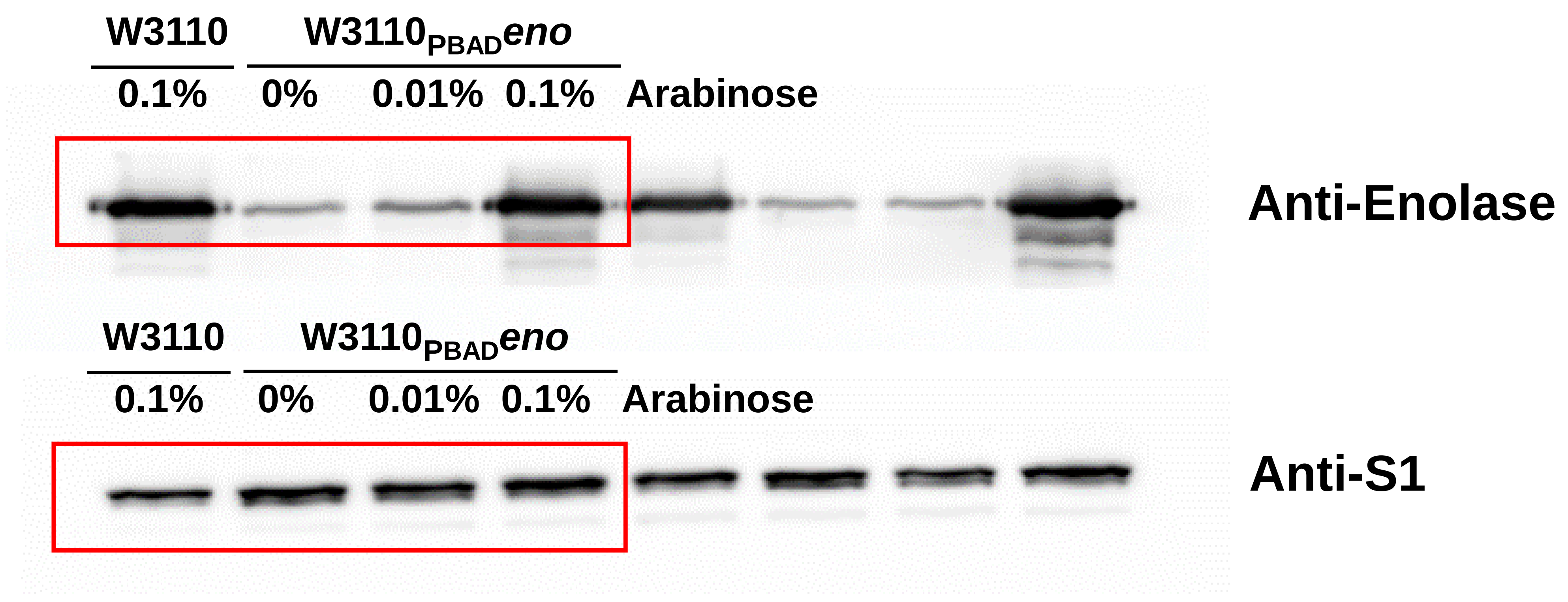

Supplement: Supplementary file 1 — Supplementary information [file 41598_2019_53883_MOESM1_ESM.pdf]
